# Supplementary material for: Orexin receptors 1 and 2 in serotonergic neurons differentially regulate peripheral glucose metabolism in obesity
Source: Nat Commun. 2021 Sep 2;12:5249. doi: 10.1038/s41467-021-25380-2 (PMC8413382; doi:10.1038/s41467-021-25380-2)
Supplement: Supplementary file 1 — Supplementary Information [file 41467_2021_25380_MOESM1_ESM.pdf]

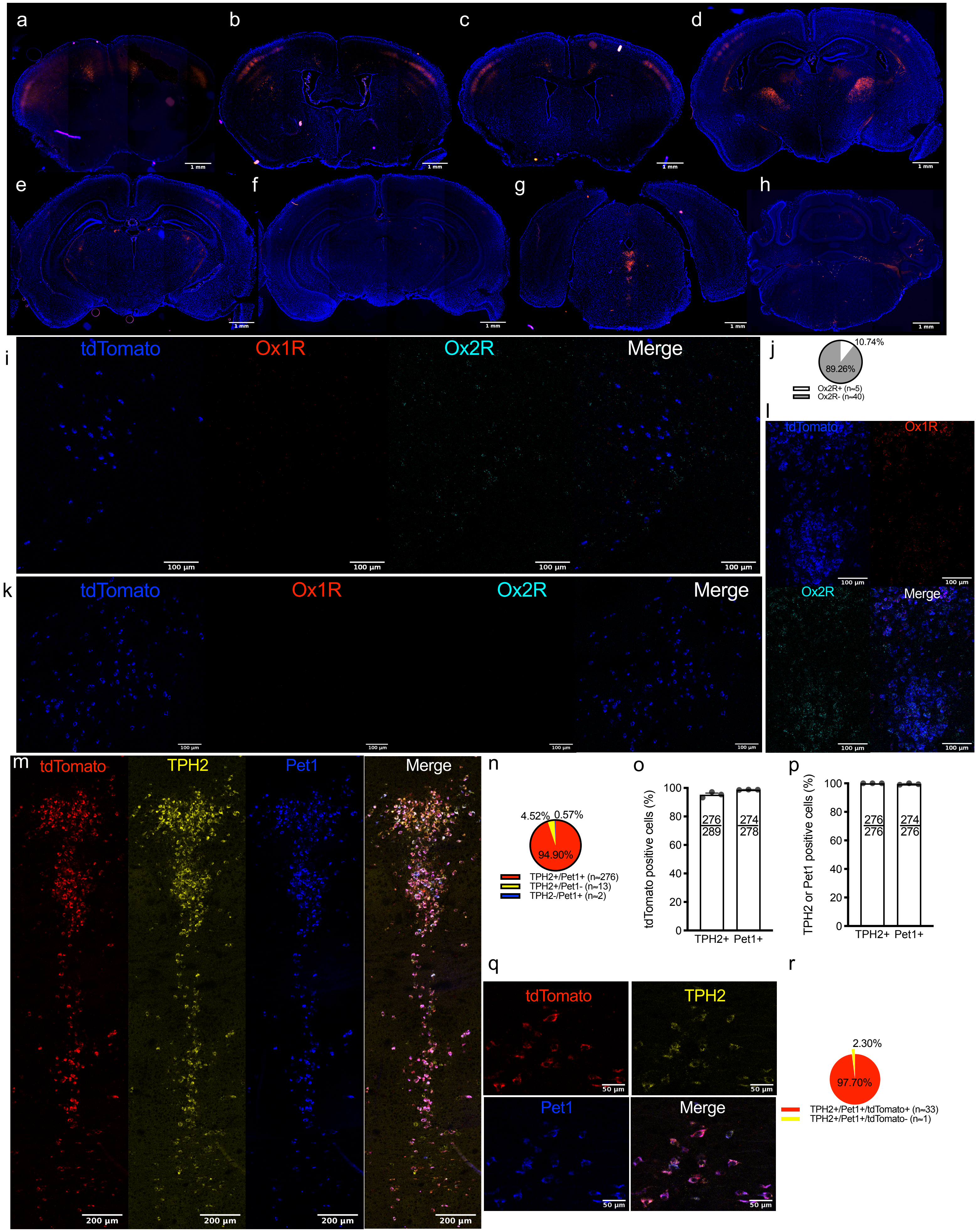


**Supplementary Figure 1. Confirmation of Cre expression in serotonergic neurons using SERT$^{tdTomato}$ mice.**

**(a-h)** Representative images of tdTomato autofluorescence in the whole brain. Blue, dapi; red, tdTomato. **(i)** Representative images of RNAscope *in situ* hybridization of tdTomato, Ox1R and Ox2R in tdTomato positive areas in cortex and **(k)** ventral posteromedial (VPM) and ventral posterolateral (VPL) thalamic nuclei; **(j)** the percentage of Ox2R-expressing cells in tdTomato positive cells in cortex; **(l)** representative images of a positive control in dorsal raphe nucleus. Blue, tdTomato; red, Ox1R; cyan, Ox2R. n = 3. **(m)** Representative images of RNAscope *in situ* hybridization of tdTomato, tryptophan hydroxylase isoform 2 (TPH2) and Pet1 in dorsal and median raphe nuclei, and quantification: **(n)** co-expression pattern of TPH2 and Pet1, **(o)** percentages of tdTomato positive cells in TPH2 or Pet1 positive cells and **(p)** percentage of TPH2 or Pet1 positive cells in tdTomato positive cells; **(q)** representative images of RNAscope *in situ* hybridization in raphe pallidus, and quantification: **(r)** percentages of tdTomato positive cells in TPH2 and Pet1 positive cells. Red, tdTomato; yellow, TPH2; blue, Pet1. n = 3. Scale bar: 1 mm (a-h), 200 µm (m), 100 µm (i, k, l) or 50 µm (q). Data are represented as means ± SEM. Source data are provided as a Source Data file.

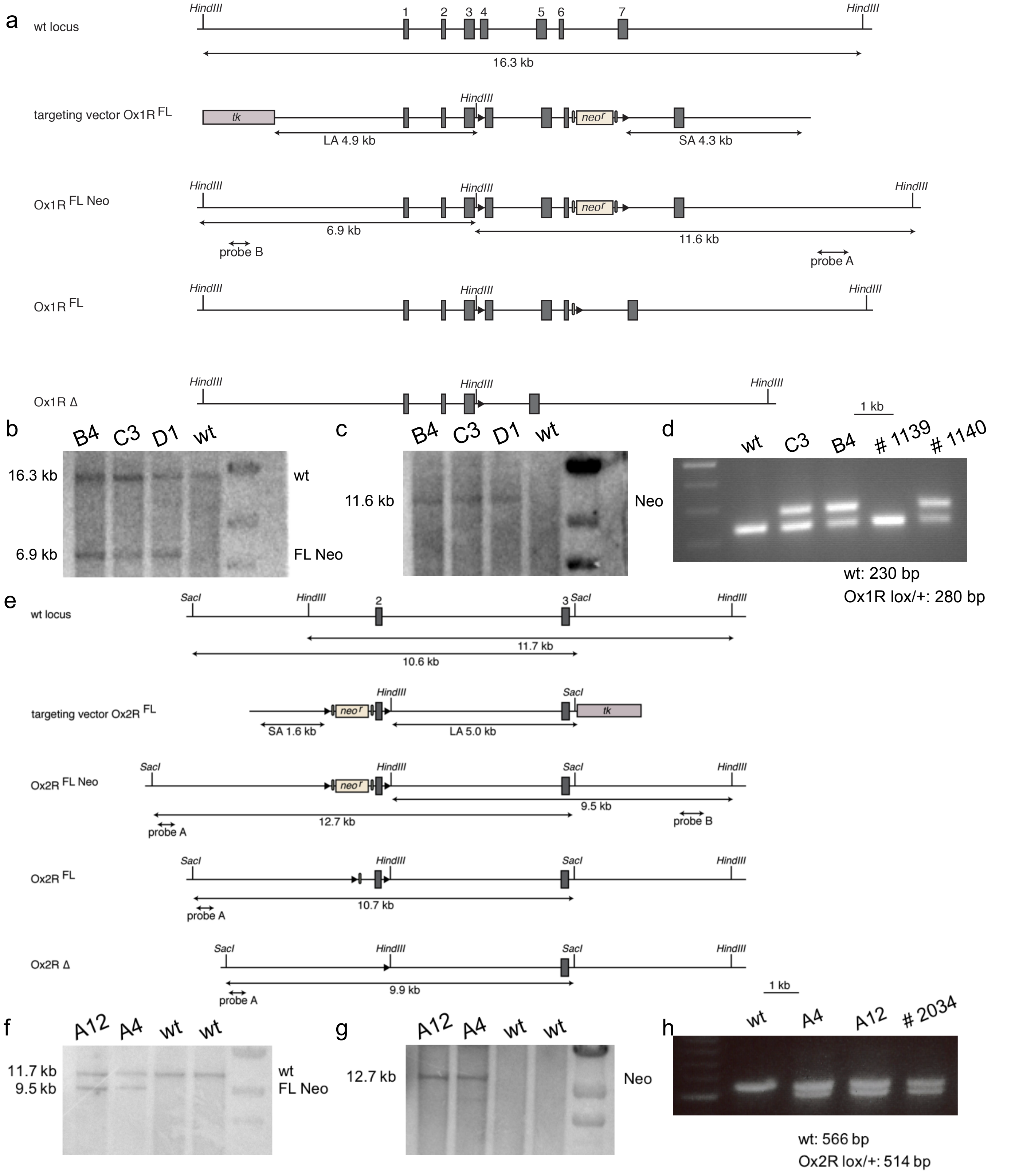


**Supplementary Figure 2. Generation of mice with a conditional Ox1R allele.**

**(a)** Targeting strategy for the generation of a conditional Ox1R allele. In Southern Blot analysis on HindIII-digested DNA using probe A/B, the wildtype Ox1R gene (wt locus) comprising exons 1-7 produces a 16.3 kb band. The Ox1R targeting vector was generated by inserting the short arm of homology (SA), the loxP-flanked exons 4,5, and 6, and the long arm of homology (LA) into the GK12TK plasmid using common cloning techniques. Homologous recombination creates the FL Neo allele, which produces a 6.9 kb fragment in Southern Blot analysis using probe B on HindIII-digested DNA. **(b)** B4, C3, and D1 ES cell clones were identified and verified by Southern Blot analysis using probe B. **(c)** Southern Blot analysis of the same clones hybridized with the probe corresponding to the neomycin resistance gene for verification of single integration. **(d)** Tail-DNA (#1139, 1140) and C3/B4 ES cell clone DNA PCR analysis with primers flanking the 3'loxP-site confirming co-integration of the loxP-site. **(e)** Targeting strategy for the generation of a conditional Ox2R allele. In Southern Blot analysis on HindIII-digested DNA using probe B, the wt Ox2R gene (wt locus) comprising exons 2 and 3 produces an 11.7 kb band. The Ox2R targeting vector was generated by inserting the short arm of homology, the loxP-flanked exon 2, and the long arm of homology into the GK12TK plasmid using common cloning techniques. Homologous recombination creates the FL Neo allele, which produces a 9.5 kb fragment in Southern Blot analysis using probe B on HindIII-digested DNA. **(f)** A4 and A12 ES cell clones were identified and verified by Southern Blot analysis using probe B. **(g)** Southern Blot analysis of the same clones hybridized with the probe corresponding to the neomycin resistance gene for verification of single integration. **(h)** Tail-DNA (#2034) and A4/A12 ES cell clone DNA PCR analysis with primers flanking the 3'loxP-site confirming co-integration of the loxP-site. Replicates ($n \geq 2$) of all images were sucessful.

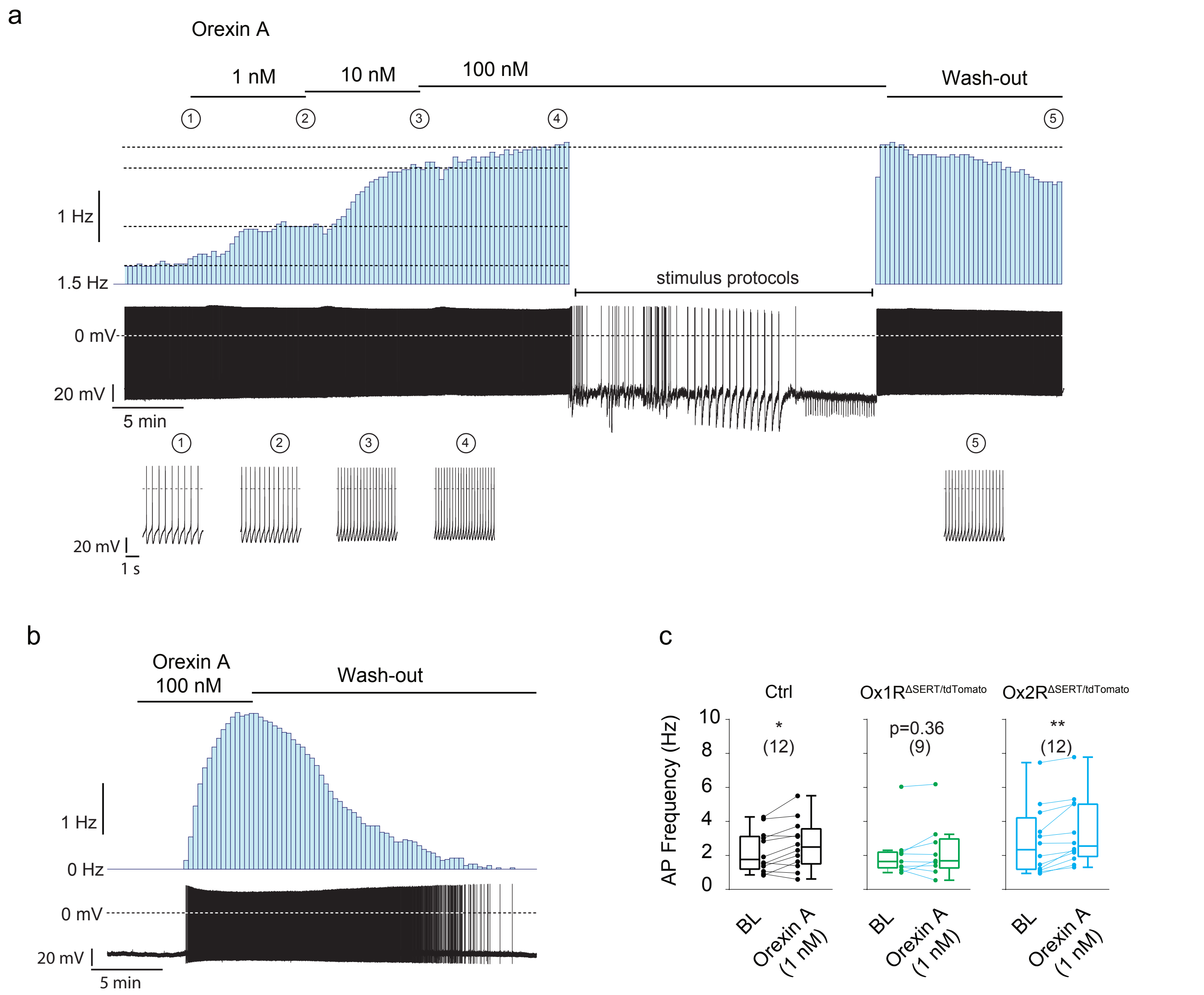


**Supplementary Figure 3. Activation of dorsal raphe-dorsal (DRD) serotonergic neurons by orexin.**

**(a, b)** Orexin A modulation of two individual DRD serotonergic neurons. **(a)** Firing rate before, during, and after application of increasing orexin A concentrations (1 nM, 10 nM, 100 nM) (top) and the corresponding original recording (middle). Bottom: Original recordings with higher time resolution at the indicated time points 1–5. **(b)** Firing rate (top) and corresponding original recording (bottom) before, during, and after application of orexin A (100 nM). This recording shows a single, short orexin A application to demonstrate the reversibility of the orexin A effect. It was not included in the quantitative analysis of concentration-dependent effects. Bin width in (a) and (b) is 20 s. **(c)** Boxplots showing the effect of orexin A (1 nM) on the action potential frequency of DRD serotonergic neurons from control, $Ox1R^{\Delta SERT/tdTomato}$, and $Ox2R^{\Delta SERT/tdTomato}$ mice. Mean firing frequencies were compared using paired two-tailed student's t-test. Orexin A application: Ctrl, $p = 0.018$, $n = 12$; $Ox1R^{\Delta SERT/tdTomato}$: $p = 0.36$, $n = 9$; $Ox2R^{\Delta SERT/tdTomato}$: $p = 0.0016$, $n = 12$. In the box plots, horizontal lines show the medians of the data. Boxes indicate the 25th and 75th percentiles. The whiskers were calculated according to the 'Tukey' method. * $p < 0.05$, ** $p < 0.01$. n values are given in brackets. Abbreviation: baseline (BL). Source data are provided as a Source Data file.

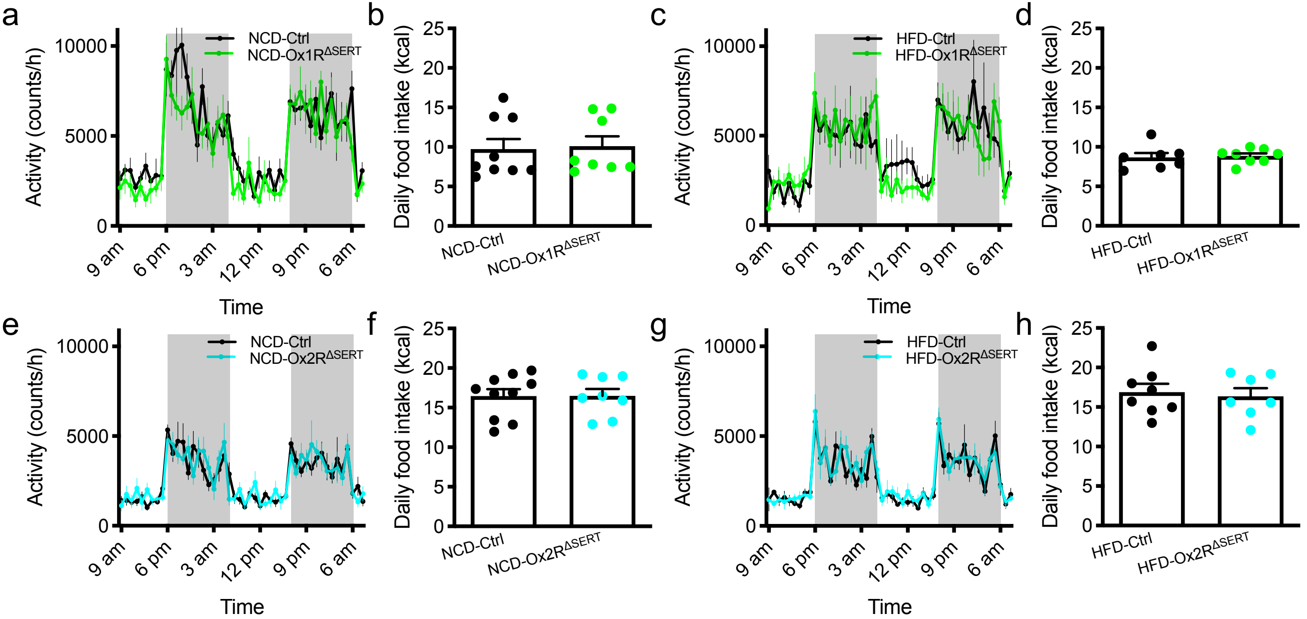


**Supplementary Figure 4. Unaltered energy homeostasis and locomotor activity in Ox1R$^{\Delta SERT}$ and Ox2R$^{\Delta SERT}$ mice on HFD.** **(a)** Locomotor activity and **(b)** daily food intake of Ox1R$^{\Delta SERT}$ mice fed a normal chow diet (NCD). NCD-Ctrl, n = 9; NCD-Ox1R$^{\Delta SERT}$, n = 9 (a) or 8 (b). **(c)** Locomotor activity and **(d)** daily food intake of Ox1R$^{\Delta SERT}$ mice fed a high-fat diet (HFD). HFD-Ctrl, n = 7; HFD-Ox1R$^{\Delta SERT}$, n = 9 (c) or 8 (d). **(e)** Locomotor activity and **(f)** daily food intake of Ox2R$^{\Delta SERT}$ mice fed a NCD. NCD-Ctrl, n = 10; NCD-Ox2R$^{\Delta SERT}$, n = 8. **(g)** Locomotor activity and **(h)** daily food intake of Ox2R$^{\Delta SERT}$ mice fed a HFD. HFD-Ctrl, n = 8; HFD-Ox2R$^{\Delta SERT}$, n = 7. Grey boxes in the background indicate the dark phase. Data are represented as means ± SEM. Source data are provided as a Source Data file.

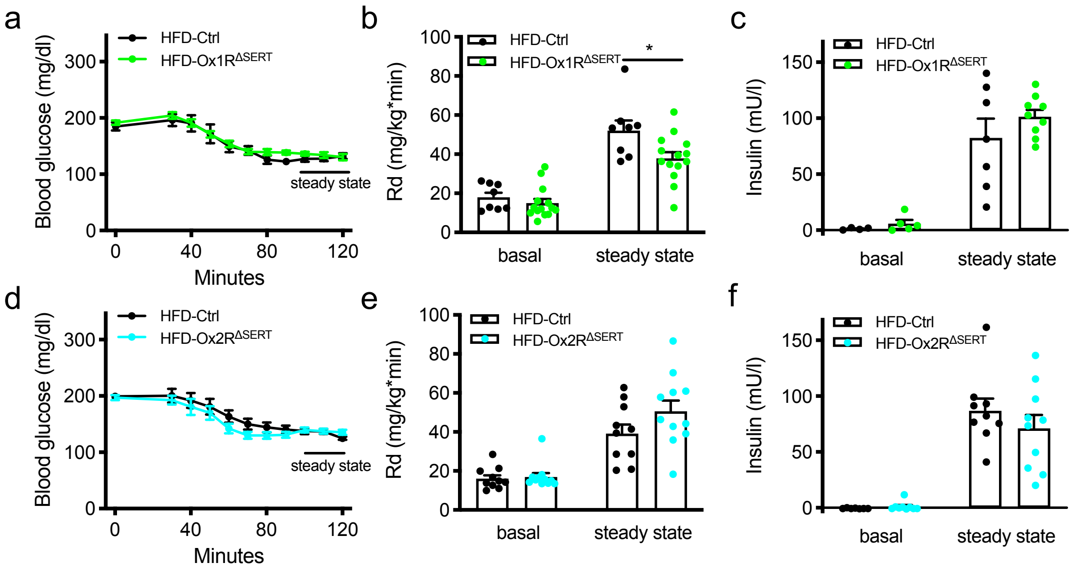


**Supplementary Figure 5. Reduced glucose clearance in Ox1R$^{ΔSERT}$ mice but not in Ox2R$^{ΔSERT}$ mice fed a HFD.**
**(a)** Blood glucose levels during hyperinsulinemic-euglycemic clamp analysis of Ox1R$^{ΔSERT}$ mice. HFD-Ctrl, n = 8; HFD-Ox1R$^{ΔSERT}$, n = 14. **(b)** Rate of glucose disappearance (Rd) at the basal and steady state of the clamp analysis of Ox1R$^{ΔSERT}$ mice. HFD-Ctrl, n = 8; HFD-Ox1R$^{ΔSERT}$, n = 14. p = 0.023 (steady state). **(c)** Human insulin concentrations in serum of control and Ox1R$^{ΔSERT}$ mice. HFD-Ctrl, n = 4 (basal) or 7 (steady state); HFD-Ox1R$^{ΔSERT}$, n = 5 (basal) or 9 (steady state). **(d)** Blood glucose levels during hyperinsulinemic-euglycemic clamp analysis of Ox2R$^{ΔSERT}$ mice. HFD-Ctrl, n = 10; HFD-Ox2R$^{ΔSERT}$, n = 11. **(e)** Rate of glucose disappearance (Rd) at the basal and steady state of the clamp analysis of Ox2R$^{ΔSERT}$ mice. HFD-Ctrl, n = 10; HFD-Ox2R$^{ΔSERT}$, n = 11. **(f)** Human insulin concentrations in serum of control and Ox2R$^{ΔSERT}$ mice. HFD-Ctrl, n = 7 (basal) or 9 (steady state); HFD-Ox2R$^{ΔSERT}$, n = 9 (basal) or 10 (steady state). Data are represented as means ± SEM. *$p < 0.05$, as determined by unpaired two-tailed Student's t-test. Source data are provided as a Source Data file.

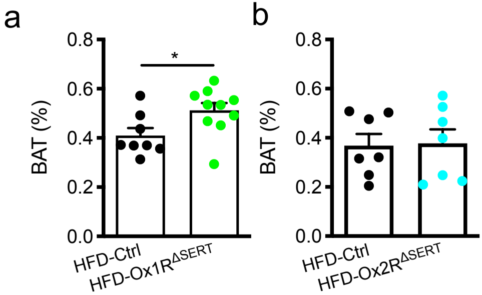


**Supplementary Figure 6. Increased brown adipose tissue (BAT) weight in Ox1R$^{\Delta SERT}$ mice but not in Ox2R$^{\Delta SERT}$ mice fed a high-fat diet (HFD).**

**(a)** Percentages of BAT weight relative to body weight in Ox1R$^{\Delta SERT}$ mice fed a HFD. HFD-Ctrl, n = 8; HFD-Ox1R$^{\Delta SERT}$, n = 10. p = 0.031. **(b)** Percentages of BAT weight relative to body weight in Ox2R$^{\Delta SERT}$ mice fed a HFD. n = 7. Data are represented as means ± SEM. *$p < 0.05$, as determined by unpaired two-tailed Student's t-test. Source data are provided as a Source Data file.

tdTomato in magenta, orexin in green and dapi in blue

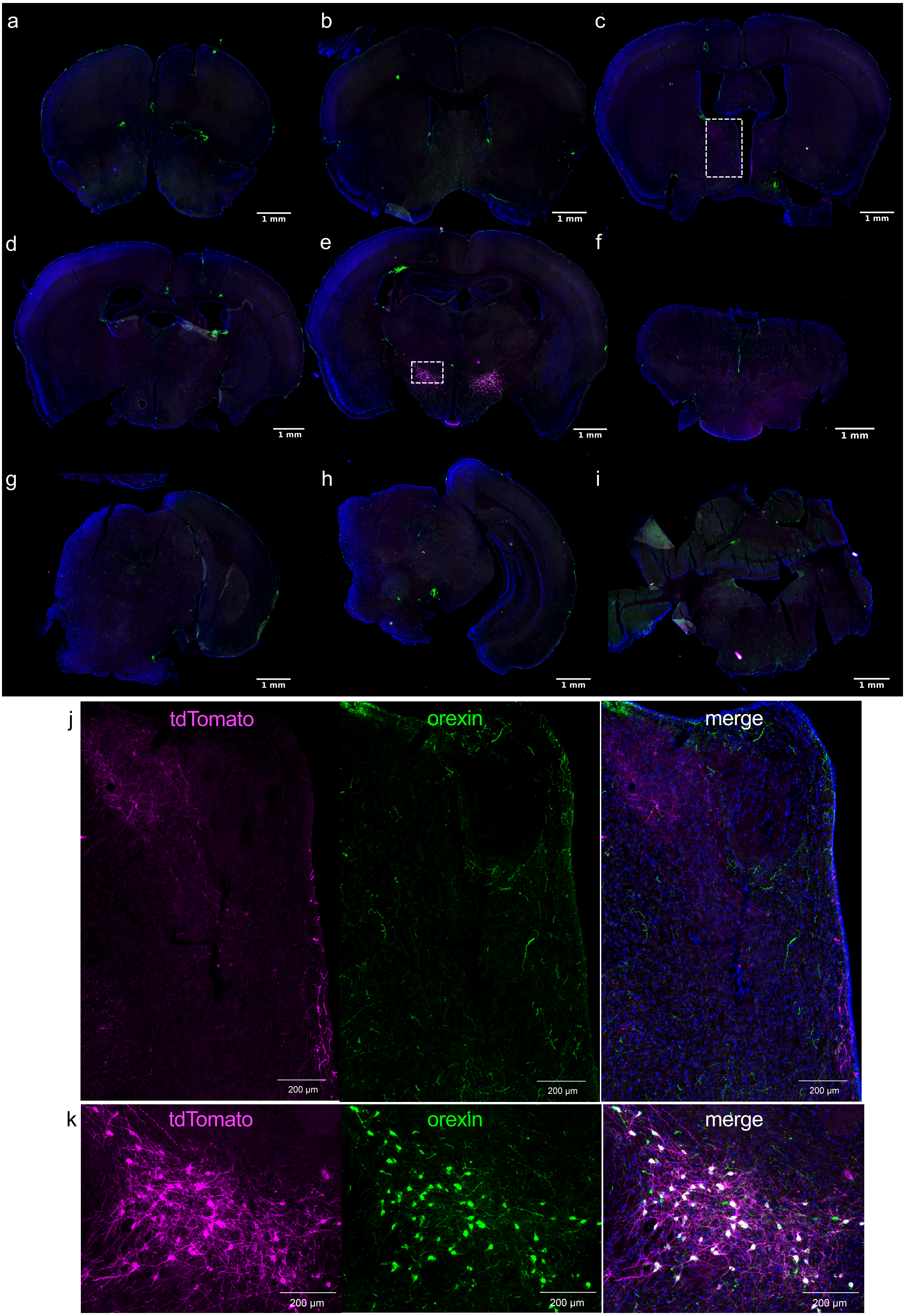


**Supplementary Figure 7. Analysis of Cre expression in orexin neurons using orexin$^{ChR2\text{-}tdTomato}$ mice.**
**(a-i)** Representative images of immunostaining of orexin A and tdTomato in the whole brain. n = 3. **(j)** Amplified images of marked areas in (c) and **(k)** in (e). n = 3. Magenta, tdTomato; green, orexin; blue, dapi. Scale bar: 1 mm (a-i), 200 μm (j, k).

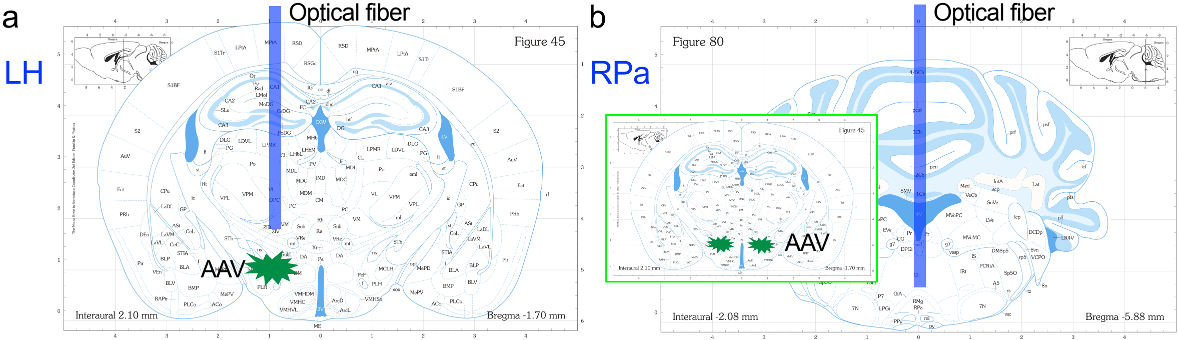


**Supplementary Figure 8. Schematic drawing of the optogenetic strategy.**

**(a)** Scheme of adeno-associated virus (AAV) injection in lateral hypothalamus (LH) and optical fiber implantation above orexin neurons. **(b)** Scheme of AAV injection in LH (in green box) and optical fiber implantation above raphe pallidus (RPa). The images showing mouse brain in stereotaxic coordinates are from the mouse brain atlas[1].

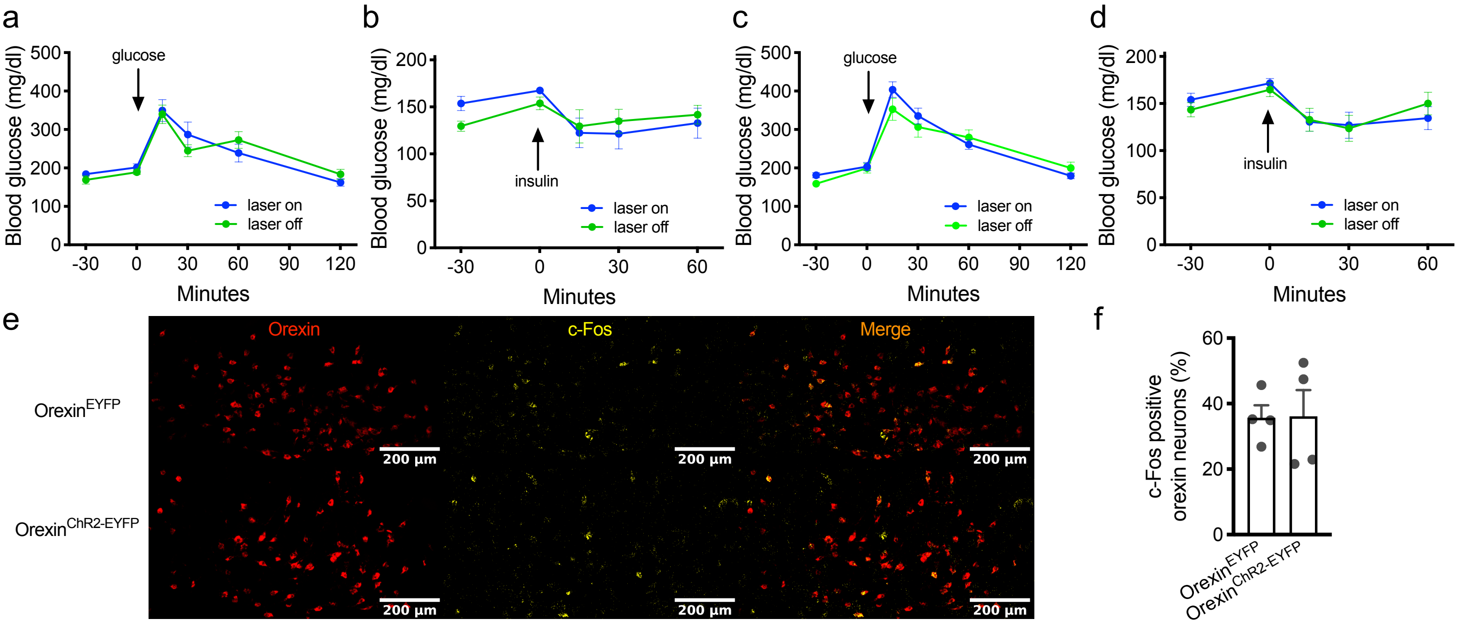


**Supplementary Figure 9. Laser illumination fails to affect glucose metabolism in mice injected with pAAV-EYFP (Orexin$^{EYFP}$).**
**(a)** Glucose tolerance test and **(b)** insulin tolerance test of Orexin$^{EYFP}$ mice with (laser on) or without (laser off) laser illumination in lateral hypothalamus (LH). n = 8. **(c)** Glucose tolerance test and **(d)** insulin tolerance test of Orexin$^{EYFP}$ mice with or without laser illumination in raphe pallidus (RPa). n = 8. **(e)** Representative images of RNAscope *in situ* hybridization of c-Fos and orexin in LH and **(f)** the quantification of percentages of c-Fos positive neurons in orexin neurons of mice after optical stimulation of orexinergic fibers in RPa. n = 4. Red, orexin; yellow, c-Fos. Scale bar: 200 µm. Data are represented as means ± SEM. Source data are provided as a Source Data file.

## Supplementary Table 1: Primers for real-time qPCR

| Gene symbol | Description | TaqMan probes |
|---|---|---|
| *Ucp1* | uncoupling protein 1 (mitochondrial, proton carrier) | Mm01244861_m1 |
| *Ppargc1a* | peroxisome proliferative activated receptor, gamma, coactivator 1 alpha | Mm01208835_m1 |
| *Acox3* | acyl-Coenzyme A oxidase 3, pristanoyl | Mm00446122_m1 |
| *Vegfa* | vascular endothelial growth factor A | Mm00437304_m1 |
| *Cycs* | cytochrome c, somatic | Mm01621048_s1 |
| *Mfn1* | mitofusin 1 | Mm00612599_m1 |
| *Mfn2* | mitofusin 2 | Mm00500120_m1 |
| *Dnm1l* | dynamin 1-like | Mm01342903_m1 |
| *Fis1* | fission 1 (mitochondrial outer membrane) homolog (yeast) | Mm00481580_m1 |
| *Mff* | mitochondrial fission factor | Mm01273401_m1 |
| *Gapdh* | glyceraldehyde-3-phosphate dehydrogenase | Mm99999915_g1 |

**Supplementary References**

1 Franklin, K. B. J. & Paxinos, G. *The mouse brain in stereotaxic coordinates*. 3rd edn, (Elsevier/Academic Press, 2007).
