# Supplementary material for: Orexin receptors 1 and 2 in serotonergic neurons differentially regulate peripheral glucose metabolism in obesity
Source: Nat Commun. 2021 Sep 2;12:5249. doi: 10.1038/s41467-021-25380-2 (PMC8413382; doi:10.1038/s41467-021-25380-2)
Supplement: Supplementary file 3 — Reporting Summary [file 41467_2021_25380_MOESM3_ESM.pdf]

nature research

Corresponding author(s): A. Christine Hausen

Last updated by author(s): Jun 24, 2021

Reporting Summary

Nature Research wishes to improve the reproducibility of the work that we publish. This form provides structure for consistency and transparency in reporting. For further information on Nature Research policies, see our Editorial Policies and the Editorial Policy Checklist.

Statistics

For all statistical analyses, confirm that the following items are present in the figure legend, table legend, main text, or Methods section.

n/a	Confirmed	
☐	☒	The exact sample size (*n*) for each experimental group/condition, given as a discrete number and unit of measurement
☐	☒	A statement on whether measurements were taken from distinct samples or whether the same sample was measured repeatedly
☐	☒	The statistical test(s) used AND whether they are one- or two-sided<br>*Only common tests should be described solely by name; describe more complex techniques in the Methods section.*
☒	☐	A description of all covariates tested
☐	☒	A description of any assumptions or corrections, such as tests of normality and adjustment for multiple comparisons
☐	☒	A full description of the statistical parameters including central tendency (e.g. means) or other basic estimates (e.g. regression coefficient) AND variation (e.g. standard deviation) or associated estimates of uncertainty (e.g. confidence intervals)
☐	☒	For null hypothesis testing, the test statistic (e.g. *F*, *t*, *r*) with confidence intervals, effect sizes, degrees of freedom and *P* value noted<br>*Give P values as exact values whenever suitable.*
☒	☐	For Bayesian analysis, information on the choice of priors and Markov chain Monte Carlo settings
☒	☐	For hierarchical and complex designs, identification of the appropriate level for tests and full reporting of outcomes
☒	☐	Estimates of effect sizes (e.g. Cohen's *d*, Pearson's *r*), indicating how they were calculated

Our web collection on statistics for biologists contains articles on many of the points above.

Software and code

Policy information about availability of computer code

Data collection	Image acquisition software: Leica TCS SP8 , Zeiss EM109, JEOL JEM 2100Plus;<br><br>indirect calorimetry data acquisition was carried out using TSE Phenomaster versions 6.2.5 and above;<br><br>electrophysiological data were recorded using a micro1410 data acquisition interface and Spike 2 (version 7) (both from CED, Cambridge, UK); during calcium imaging, the camera and polychromator were controlled by the software Zen pro, including the module ‘Physiology’ (2012 blue edition, Zeiss). The emitted fluorescence was detected through a 500-550 nm bandpass filter (BP525/50), and data were acquired using 4x4 on-chip binning.
Data analysis	Details are provided in the Methods section. The main software used are as follows:<br><br>For the RNA-sequencing results, we applied the community-curated nfcore rnaseq analysis pipeline version 1.4. Codes are available on Github. The gene-level quantification was carried out using Salmon 0.14.1 using the reference genome GRCm38. The differential gene expression analysis was done using the DESeq2 1.26.0 R package. A gene-ontology term analysis was carried out using the clusterProfiler 3.14.364 R package.<br><br>We also used Spike2 (version 7; Cambridge Electronic Design Ltd., Cambridge, UK), Igor Pro 6 (Wavemetrics, Portland, OR, USA), Halo 2.0 (Indica Labs), Graphpad Prism (version 8.0; Graphpad Software Inc., La Jolla, CA, USA) and Image J/FIJI (version 1.50d and 1.53a).<br><br>The source code for analysing the RNA-Seq data presented in this study are deposited on GitHub with the repository bruening-lab/Sert-Ox1R (https://github.com/bruening-lab/Sert-Ox1R).

For manuscripts utilizing custom algorithms or software that are central to the research but not yet described in published literature, software must be made available to editors and reviewers. We strongly encourage code deposition in a community repository (e.g. GitHub). See the Nature Research guidelines for submitting code & software for further information.

Data

Policy information about availability of data

All manuscripts must include a data availability statement. This statement should provide the following information, where applicable:

- Accession codes, unique identifiers, or web links for publicly available datasets
- A list of figures that have associated raw data
- A description of any restrictions on data availability

The RNA-Seq data generated in this study have been deposited in the GEO database under accession code GSE168203 (https://www.ncbi.nlm.nih.gov/geo/query/acc.cgi?acc=GSE168203). The source code for analysing the RNA-Seq data presented in this study are deposited on GitHub with the repository bruening-lab/Sert-Ox1R (https://github.com/bruening-lab/Sert-Ox1R).

Publicly available datasets used in this study include GRCm38 dataset (https://www.ncbi.nlm.nih.gov/assembly/GCF_000001635.20) and "scRNA-seq_huang2019" (https://doi.org/10.7910/DVN/QB5CC8).

The source data underlying Figs. 1c-i,k-m, 2e-h, 3b,c,e, 4b-r, 5a-f, 6a-f, 7c-l,n-p, 8b,d,e,g, 9d,e,g and Supplementary Figs. 1j,n,o,p,r, 3c, 4a-h, 5a-f, 6a,b, 9a-d,f are provided as a Source Data file.

Raw data that support the findings of this study are available from the corresponding author upon reasonable request.

There are no restrictions on data availability.

Field-specific reporting

Please select the one below that is the best fit for your research. If you are not sure, read the appropriate sections before making your selection.

[x] Life sciences [ ] Behavioural & social sciences [ ] Ecological, evolutionary & environmental sciences

For a reference copy of the document with all sections, see nature.com/documents/nr-reporting-summary-flat.pdf

Life sciences study design

All studies must disclose on these points even when the disclosure is negative.

Sample size	No statistical methods were used to pre-determine sample sizes and the sample sizes in the study were chosen based on experience from previous in house studies of metabolic phenotyping and to balance between the ability to detect significance differences while reducing the number of animals used. They are similar to those reported previously: Brandt et al. (2018), Vogt et al. (2015), Konner et al. (2007).
Data exclusions	No data was excluded.
Replication	Each mouse represents a biological replicate (n >= 3) and the numbers are mentioned in each figure and/or figure legends. For images analyzed in this study, at least 2 technical replicates were performed for each mouse and the average values were taken for statistical analysis. For images without statistic analysis, biological replicates are more than 3, which are listed in figure legends, and technical replicates are more than 2. All attempts for replicates are successful. Findings in this study are reproducible and can be replicated.
Randomization	The control and conditional knockout mice were litter-mates and assigned to corresponding groups by genotypes. For virus injection experiments, litter-mates were assigned to inject control or ChR2 virus randomly.
Blinding	The data collection and analysis was performed when being blind to genotypes.

Reporting for specific materials, systems and methods

We require information from authors about some types of materials, experimental systems and methods used in many studies. Here, indicate whether each material, system or method listed is relevant to your study. If you are not sure if a list item applies to your research, read the appropriate section before selecting a response.

Materials & experimental systems

n/a	Involved in the study
	x Antibodies
x	Eukaryotic cell lines
x	Palaeontology and archaeology
	x Animals and other organisms
x	Human research participants
x	Clinical data
x	Dual use research of concern

Methods

n/a	Involved in the study
x	ChIP-seq
x	Flow cytometry
x	MRI-based neuroimaging

Antibodies

Antibodies used

Primary antibodies:
pSer473-Akt ((#4060, rabbit mAb, Cell Signaling Technology), Akt (#4685, rabbit mAb, Cell signaling Technology), G6Pase (sc-25840, rabbit pAb, Santa Cruz Biotechnology), UCP-1 (sc-6528, goat pAb, Santa Cruz Biotechnology), Tom20 (sc-17764, mouse mAb, Santa Cruz Biotechnology), OPA1 (612607, mouse mAb, BD Biosciences), MFN1 (ab57602, mouse mAb, Abcam), MFN2 (ab56889, mouse mAb, Abcam), DRP1 (#8570, rabbit mAb, Cell Signaling Technology), MFF (17090-1-AP, rabbit pAb, ProteintechR), FIS1 (10956-1-AP,rabbit pAb, ProteintechR), complex I-V subunits in the respiratory chain (Total OXPHOS Rodent WB Antibody Cocktail, containing 5 mouse mAbs, ab110413, Abcam), Calnexin (208880, rabbit pAb, CalbiochemR), orexin-A (sc-8070, goat pAb, Santa Cruz Biotechnology), GFP (ab13970, chicken pAb, Abcam),), TPH2 (#51124, rabbit mAb, Cell Signaling Technology) and serotonin (S5545, rabbit pAb, Sigma-Aldrich).

Secondary antibodies:
Peroxidase conjugate goat anti-rabbit IgG (A6154), goat anti-mouse IgG (A4416) and mouse anti-goat IgG (A9452) were purchased from Sigma-Aldrich. Alexa Fluro 594 donkey anti-goat, FITC donkey anti-chicken, Alexa Fluro 488 donkey anti-rabbit, Alexa Fluro 647 donkey anti-rabbit (a11058, sa 1-7200, a21206 and a31573, respectively) were purchased from Invitrogen.

Validation

As validated by the company.

pSer473-Akt ((#4060, rabbit mAb, Cell Signaling Technology): https://www.cellsignal.de/products/primary-antibodies/phospho-akt-ser473-d9e-xp-rabbit-mab/4060;

Akt (#4685, rabbit mAb, Cell signaling Technology): https://www.cellsignal.de/products/primary-antibodies/akt-pan-11e7-rabbit-mab/4685;

G6Pase (sc-25840, rabbit pAb, Santa Cruz Biotechnology): https://datasheets.scbt.com/sc-25840.pdf;

UCP-1 (sc-6528, goat pAb, Santa Cruz Biotechnology): https://datasheets.scbt.com/sc-6528.pdf;

Tom20 (sc-17764, mouse mAb, Santa Cruz Biotechnology): https://datasheets.scbt.com/sc-17764.pdf;

OPA1 (612607, mouse mAb, BD Biosciences), https://www.bdbiosciences.com/en-us/products/reagents/microscopy-imaging-reagents/immunofluorescence-reagents/purified-mouse-anti-opa1.612607;

MFN1 (ab57602, mouse mAb, Abcam): https://www.abcam.com/mitofusin-2--mitofusin-1-antibody-3c9-ab57602.html;

MFN2 (ab56889, mouse mAb, Abcam): https://www.abcam.com/mitofusin-2-antibody-6a8-ab56889.html;

DRP1 (#8570, rabbit mAb, Cell Signaling Technology): https://www.cellsignal.de/products/primary-antibodies/drp1-d6c7-rabbit-mab/8570;

MFF (17090-1-AP, rabbit pAb, ProteintechR): https://www.ptglab.com/products/MFF-Antibody-17090-1-AP.htm;

FIS1 (10956-1-AP,rabbit pAb, ProteintechR): https://www.ptglab.com/products/FIS1-Antibody-10956-1-AP.htm;

complex I-V subunits in the respiratory chain (Total OXPHOS Rodent WB Antibody Cocktail, containing 5 mouse mAbs, ab110413, Abcam): https://www.abcam.com/total-oxphos-rodent-wb-antibody-cocktail-ab110413.html;

Calnexin (208880, rabbit pAb, CalbiochemR): https://www.merckmillipore.com/DE/de/product/Anti-Calnexin-C-Terminal-575-593-Rabbit-pAb,EMD_BIO-208880?ReferrerURL=https%3A%2F%2Fwww.google.com%2F;

orexin-A (sc-8070, goat pAb, Santa Cruz Biotechnology): https://www.scripps.edu/martin-fardon/OrxA%20Antibody%20for%20IHC.pdf;

GFP (ab13970, chicken pAb, Abcam): https://www.abcam.com/gfp-antibody-ab13970.html;

TPH2 (#51124, rabbit mAb, Cell Signaling Technology): https://www.cellsignal.de/products/primary-antibodies/tph2-d3e5i-xp-rabbit-mab/51124;

serotonin (S5545, rabbit pAb, Sigma-Aldrich): https://www.sigmaaldrich.com/DE/de/product/sigma/s5545.

Animals and other organisms

Policy information about studies involving animals; ARRIVE guidelines recommended for reporting animal research

Laboratory animals	4-21 wk old male mice from the C57BL/6N strain were used. The geneticly modified mice used in this study are as follows: tdTomato fl/fl, Slc6a4-Cre, Ox1R fl/fl, Ox2R fl/fl, ChR2-tdTomato fl/fl, Orexin-IRES-Cre, Ox1RΔSERT, Ox2RΔSERT, Sert^tdTomato, Ox1RΔSERT/tdTomato, Ox2RΔSERT/tdTomato and Orexin^ChR2-tdTomato mice. Male and females mice between 4 - 40 wk were used for breedings. Male mice between 4 - 21 wk were used for experiments. Specific age of mice for each experiment was described in Methods.
Wild animals	No wild animals were used in the study.
Field-collected samples	No field collected samples were used in the study.
Ethics oversight	All animal procedures were conducted in compliance with protocols approved by the local government authorities (Bezirksregierung Cologne, Germany) and were in accordance with National Institutes of Health guidelines. Permission to maintain and breed mice was issued by the Department for Environment and Consumer Protection - Veterinary Section, Cologne, North Rhine-Westphalia, Germany (84-02.04.2015.A335).

Note that full information on the approval of the study protocol must also be provided in the manuscript.
